# Triphenylphosphine Derivatives of Allylbenzenes Express Antitumor and Adjuvant Activity When Solubilized with Cyclodextrin-Based Formulations

**DOI:** 10.3390/ph16121651

**Published:** 2023-11-26

**Authors:** Igor D. Zlotnikov, Sergey S. Krylov, Marina N. Semenova, Victor V. Semenov, Elena V. Kudryashova

**Affiliations:** 1 Faculty of Chemistry, Lomonosov Moscow State University, Leninskie Gory, 1/3, 119991 Moscow, Russia; zlotnikovid@my.msu.ru; 2 N. D. Zelinsky Institute of Organic Chemistry RAS, 47 Leninsky Prospect, 119991 Moscow, Russia; 3 N. K. Koltzov Institute of Developmental Biology RAS, 26 Vavilov Street, 119334 Moscow, Russia

**Keywords:** PPh_3_ conjugates, allylbenzenes, apiol, synergism, A549, efflux inhibitor, anticancer activity

## Abstract

Allylbenzenes (apiol, dillapiol, myristicin and allyltetramethoxybenzene) are individual components of plant essential oils that demonstrate antitumor activity and can enhance the antitumor activity of cytotoxic drugs, such as paclitaxel, doxorubicin, cisplatin, etc. Triphenylphosphine (PPh_3_) derivatives of allylbenzenes are two to three orders of magnitude more potent than original allylbenzenes in terms of IC_50_. The inhibition of efflux pumps has been reported for allylbenzenes, and the PPh_3_ moiety is deemed to be responsible for preferential mitochondrial accumulation and the depolarization of mitochondrial membranes. However, due to poor solubility, the practical use of these substances has never been an option. Here, we show that this problem can be solved by using a complex formation with cyclodextrin (CD-based molecular containers) and polyanionic heparin, stabilizing the positive charge of the PPh_3_ cation. Such containers can solubilize both allylbenzenes and their PPh_3_ derivatives up to 0.4 mM concentration. Furthermore, we have observed that solubilized PPh_3_ derivatives indeed work as adjuvants, increasing the antitumor activity of paclitaxel against adenocarcinomic human alveolar basal epithelial cells (A549) by an order of magnitude (in terms of IC50) in addition to being quite powerful cytostatics themselves (IC_50_ in the range 1–10 µM). Even more importantly, CD-solubilized PPh_3_ derivatives show pronounced selectivity, being highly toxic for the A549 tumor cell line and minimally toxic for HEK293T non-tumor cells, red blood cells and sea urchin embryos. Indeed, in many cancers, the mitochondrial membrane is more prone to depolarization compared to normal cells, which probably explains the observed selectivity of our compounds, since PPh_3_ derivatives are known to act as mitochondria-targeting agents. According to the MTT test, 100 µM solution of PPh_3_ derivatives of allylbenzenes causes the death of up to 85% of A549 cancer cells, while for HEK293T non-cancer cells, only 15–20% of the cells died. The hemolytic index of the studied substances did not exceed 1%, and the thrombogenicity index was < 1.5%. Thus, this study outlines the experimental foundation for developing combined cytostatic medications, where effectiveness and selectivity are achieved through decreased concentration of the primary ingredient and the inclusion of adjuvants, which are safe or practically harmless substances.

## 1. Introduction

Modern therapeutic strategies are in some cases ineffective against bacterial infections and cancers, with such cases being most often associated with multiple drug resistance (MDR) [1,2,3,4,5,6,7,8,9,10,11]. Resistance mechanisms that reduce the likelihood of a patient’s being cured can be divided into two groups [12]: (i) cellular metabolism (transferases, topoisomerases, growth factors), which alters the mechanism of action of the drugs or interferes with their action, and (ii) a decrease in the intracellular concentration of the drug. The drug enters the intracellular medium through the transport channels of the plasma membrane, in which pump proteins (ATP-binding cassette protein [4,13]) can be expressed, pumping the drug out of the cell and thus reducing its effect [4,5,14,15,16,17]. The main member of the efflux pump family, MDR1 (P-glycoprotein [3,13,14,18]), causes the resistance of various types of tumors to chemotherapy. Bacteria also have efflux pumps (for example, NorA [4,9,19,20,21], P-glycoprotein) which cause the ineffectiveness of antibiotics. A number of substances that inhibit efflux (verapamil, reserpine, etc. [22]) are known to be rather toxic. Therefore, numerous studies are aimed at finding substances that inhibit efflux but at the same time are non-toxic.

In this regard, the non-toxic components of natural extracts and oils [23,24,25,26,27,28,29,30,31,32,33,34,35,36,37,38,39,40,41,42,43] attract attention as potential adjuvants to strengthen the main drug (antibacterial or antitumor drug) and reduce the off-target effects. The individual components of essential oils (allylbenzenes [44,45,46,47], terpenoids [48,49], terpenes [50,51,52], flavonoids [30,53,54], Thai herbs [55], etc.) have antioxidant, antibacterial, restorative and antitumor properties, and, moreover, they are effective inhibitors of efflux pumps [3,5,6,9,14,15,16,17,20,21,28,56,57,58,59,60] that cause bacterial resistance to antibiotics and the resistance of cancer cells to cytostatics. Thus, the individual components of essential oils and their modifications are potential candidates for empowered medicinal combinations. However, such substances are often lipophilic [2,11,19,25,27,50,58,61,62,63,64], which makes it difficult to use them in medical practice, so the adjuvant should be used in a molecular container, such as liposomes or a polymeric carrier. Cyclodextrins (CDs) [23,31,32,65,66,67,68,69,70,71,72,73,74,75,76] or polycations/polyanions (chitosan, polyethyleneimine, pectin, alginate, heparin, etc.) can serve as effective solubilizing containers that improve the bioavailability and pharmacological properties of the drug.

Apiol (1-allyl-2,5-dimethoxy-3,4-methylenedioxybenzene), a component of parsley oil, inhibits cytochrome P450 3A4 (IC_50_ 7.9 µM) [19,36,44,58,77,78,79,80], which metabolizes xenobiotics in the liver, reducing their bioavailability. Apiol demonstrates weak antibacterial and anticancer activities, but at the same time, it dramatically enhances the effect of antibiotics (for example, moxi- or levofloxacin) [44,77] and cytostatics (doxorubicin, paclitaxel, etc.) [49] by inhibiting P-glycoprotein.

Apiol analogues (myristicin, allyltetramethoxybenzene and dillapiol) have also demonstrated weak antitumor activity, but they have served as a booster for antitumor drugs (paclitaxel, doxorubicin, cisplatin) [49] due to the inhibition of mitochondrial enzymes [81,82], efflux pumps [49] and the increased permeability of the membrane of cancer cells [49,83]. It was previously shown that dillapiol (25–50 µM) induced G0/G1 cell cycle arrest, the activation of a number of caspases and, accordingly, the apoptosis of cancer cells, while apiol and its analogues had virtually no effect on benign epithelial cells in vitro [46]. Myristicin showed a similar but weaker effect. Recently, triphenylphosphine (PPh_3_) derivatives of allylbenzenes were suggested for research into improving their antiproliferative potency toward cancer cells taking into account their tendency to preferential mitochondrial accumulation [46]. The introduction of the PPh_3_ moiety, possessing both hydrophobic and charged properties, enhances the conjugate’s localization within the cell membrane and boosts inhibition against mitochondrial membrane enzymes. This was previously demonstrated by some of the authors of this article on a micellar model [82]. Cancer cells have an altered metabolism, in particular the dynamics of mitochondria (the PPh_3_ fragment can serve as an address label to cancer mitochondria), which provides many potential targets for cancer therapy [84,85,86].

Considering the action mechanism of PPh_3_ derivatives of allylbenzenes, we can assume their potential synergistic effect with the main drug, paclitaxel. The action mechanism of paclitaxel is based on the suppression of the normal process of dynamic reorganization of the microtubule network, which is responsible for cell division. In addition, paclitaxel induces the formation of abnormal clusters and causes the formation of multiple microtubule stars during mitosis. Paclitaxel is used as a first-line drug in the treatment of ovarian, breast, lung, cervical cancer, etc. The combination of paclitaxel + adjuvant is expected to be more effective than a single drug due to the action of different mechanisms and an increase in the bioavailability of the cytostatic.

In this paper, the key idea is to realize three main aspects to create enhanced antitumor activity: (i) a combination of the main cytostatic (paclitaxel) with an adjuvant (as efflux inhibitor) from the group of allylbenzenes, (ii) an increase in the mitochondrial bioavailability of the adjuvant by conjugating it with a PPh_3_ fragment due to the depolarization of mitochondrial membranes in cancer cells, and (iii) the use of cyclodextrins derivatives and a heparin polysaccharide matrix as molecular containers to obtain soluble forms of drugs and increase their bioavailability.

## 2. Results and Discussion

### 2.1. Article Design

The present work is aimed at developing and studying complex antitumor formulations based on three components: the main drug (paclitaxel), the adjuvant (apiol-PPh_3_ and its analogues) and the molecular container (cyclodextrins (CD) for the formation of inclusion complexes with paclitaxel and its adjuvant in the hydrophobic cavity of CD or heparin polyanion to stabilize the cationic triphenylphosphine fragment). In previous studies, the authors have shown that cytostatics (paclitaxel, doxorubicin, etc.) are enhanced by allylbenzenes, which can also act as promising anticancer drugs [49,87]. In this paper, PPh_3_ derivatives of allylbenzenes are used to enhance the effect of cytostatics due to the depolarization of mitochondrial membranes and considering their tendency to preferential mitochondrial accumulation. However, because PPh_3_ derivatives are poorly soluble, to realize their potential, it is necessary to develop the optimal container, providing an increase in allylbenzene–PPh_3_ solubility and obtaining double-drug inclusion complexes, which can provide synergism of the action of the main antibiotic and the adjuvant (cyclodextrin derivatives or anionic polysaccharides are proposed in this work). To achieve this, the following tasks were realized: (1) the spectral characterization of PPh_3_ derivatives of allylbenzenes and the study of their solubility, (2) the characterization of double-drug inclusion complexes of these compounds and paclitaxel with various cyclodextrins derivatives or heparin and the determination of dissociation constants of complexes, (3) analysis of the cytotoxic activity of the cytostatic agents alone and in the complex drug formulations against A549 using the MTT test and (4) analysis of the selectivity of the cytostatic activity and the safety of drugs for HEK293T non-cancerous cells in vitro using FTIR spectroscopy, red blood cells and sea urchin embryos in vivo safety study.

### 2.2. The Spectral Characteristics of PPh_3_ Derivatives of Allylbenzenes

**Figure 1:**
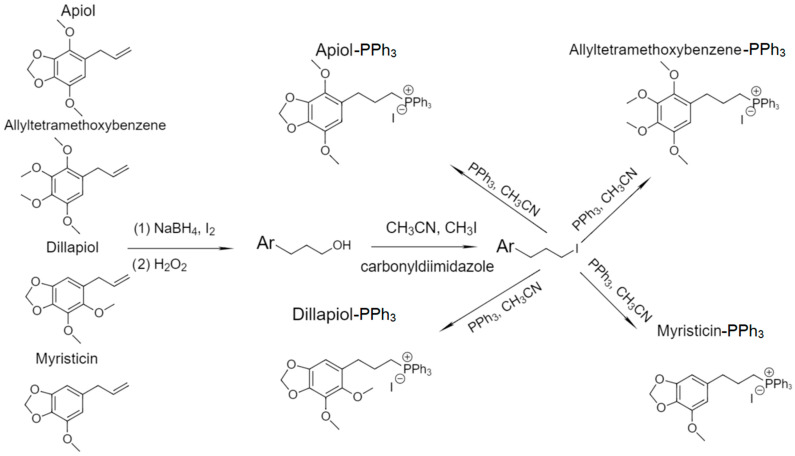
The scheme of synthesis of allylbenzenes’ PPh_3_ derivatives.

**Figure 2:**
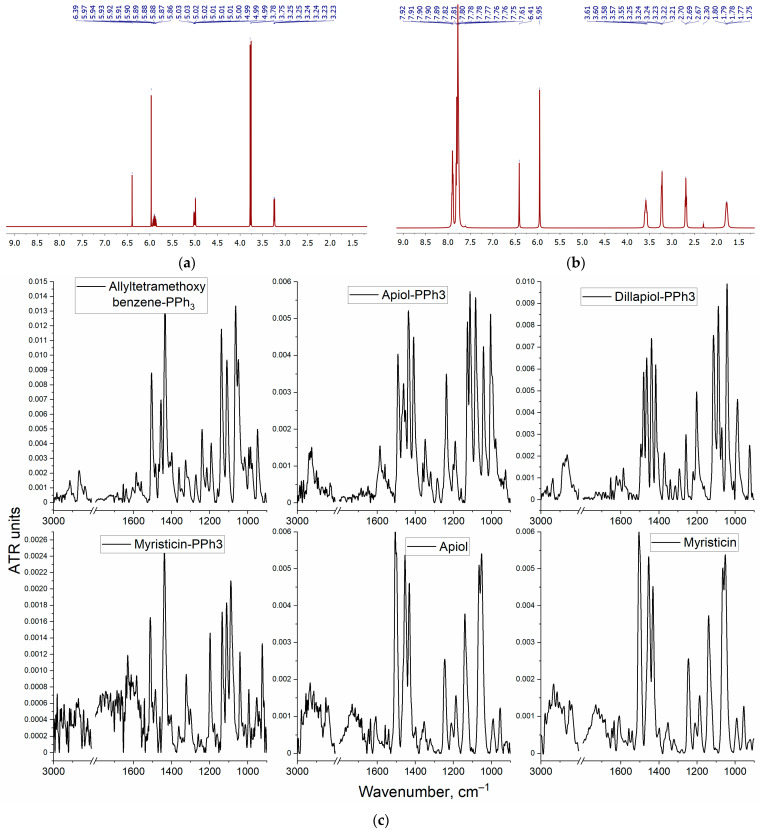
^1^H NMR spectra of (**a**) apiol; (**b**) apiol-PPh_3,_ T = 25 °C, d_6_-DMSO, 400 MHz; (**c**) FTIR spectra of apiol, apiol-PPh_3_, dillapiol-PPh_3_, myristicin, myristicin-PPh_3_ and allyltetramethoxybenzene-PPh_3_, PBS, T = 22 °C.

Allylbenzenes (apiol, myristicin, etc.) have a number of important biological activities, including experimental prerequisites to be synergists (enhancers) of the action of cytotoxic drugs. To increase the bioavailability of allylbenzenes, the modified form of allylbenzenes with a PPh_3_ fragment (Figure 1) was obtained according to the methodology described recently [46]. Confirmation of the success of synthesis follows from NMR and FTIR spectroscopy data (Figure 2 and Figure S1, Table 1). The original substances (apiol and analogues) are characterized by the main signals: aromatic protons (6 and 6.5 ppm), protons at the double bond of the allyl group (5 ppm), protons of methoxy groups and/or methylene bridges (3.3–4 ppm). After the introduction of the PPh_3_ residue into these molecules, the proton signals of the allyl group double bond disappear, but the proton signals of phenyl substituents (7.6–8.1 ppm) as well as the alkyl spacer (1.7–3 ppm) appear. In the FTIR spectra (Figure 2c) of the initial allylbenzenes, the most significant are the oscillation bands C=C 1660 cm^−1^ (allyl group) and 1450–1550 cm^−1^ (aromatic system). After the modification of allylbenzenes with PPh_3_, the peak of the oscillations of the C=C allyl group disappears, but peaks corresponding to the deformation fluctuations of the C–H triphenylphosphine fragment (1400–1480 cm^−1^) and fluctuations of C=C bonds (1500–1600 cm^−1^) appear.

### 2.3. Solubility of PPh_3_-Modified Allylbenzenes Adjuvants and Complex Formation with Cyclodextrins and Heparin

As already mentioned, PPh_3_ derivatives are poorly soluble, so to realize its potential, the formation of the inclusion complexes with cyclodextrins (CDs) (with an external hydrophilic shell and an internal hydrophobic cavity) or with heparin is suggested. This can provide the possibility of using aromatic adjuvants (which are otherwise not applicable) as an antitumor agent and to obtain an efficient combined antitumor formulation. CDs and heparin can protect the substance from destruction and inactivation, increase the half-life, and in addition, due to adsorption on cell membranes, increase the membrane permeability [49,77]. 

Loading both the main cytostatic agent and its adjuvants (apiol-PPh_3_ and its analogues) into molecular containers is suggested as a perspective approach to increase the solubility of substances in aqueous solutions, to increase the bioavailability, and consequently, improve the effectiveness of the antitumor formulation. Previously, we studied allylbenzenes as independent antitumor preparations and adjuvants to paclitaxel, where we obtained soluble forms due to complex formation with M-β-CD [77] (otherwise, these substances cannot be used at all due to insolubility and oil–water phase separation). Here, we suggest using cyclodextrins or non-cyclic polysaccharide for the preparation of soluble formulations of triphenylphosphine derivatives. We consider M-β-CD, which was efficient for allylbenzenes solubilization, as well as γ-CD, which has a larger size of the inner cavity. We have chosen heparin as a polyanion to form electrostatic complexes with positively charged PPh_3_. In addition, heparin as an antithrombotic agent in the tumor microenvironment could have an additional therapeutic effect, since the tumor’s development is a thrombosis-associated process.

**Figure 3:**
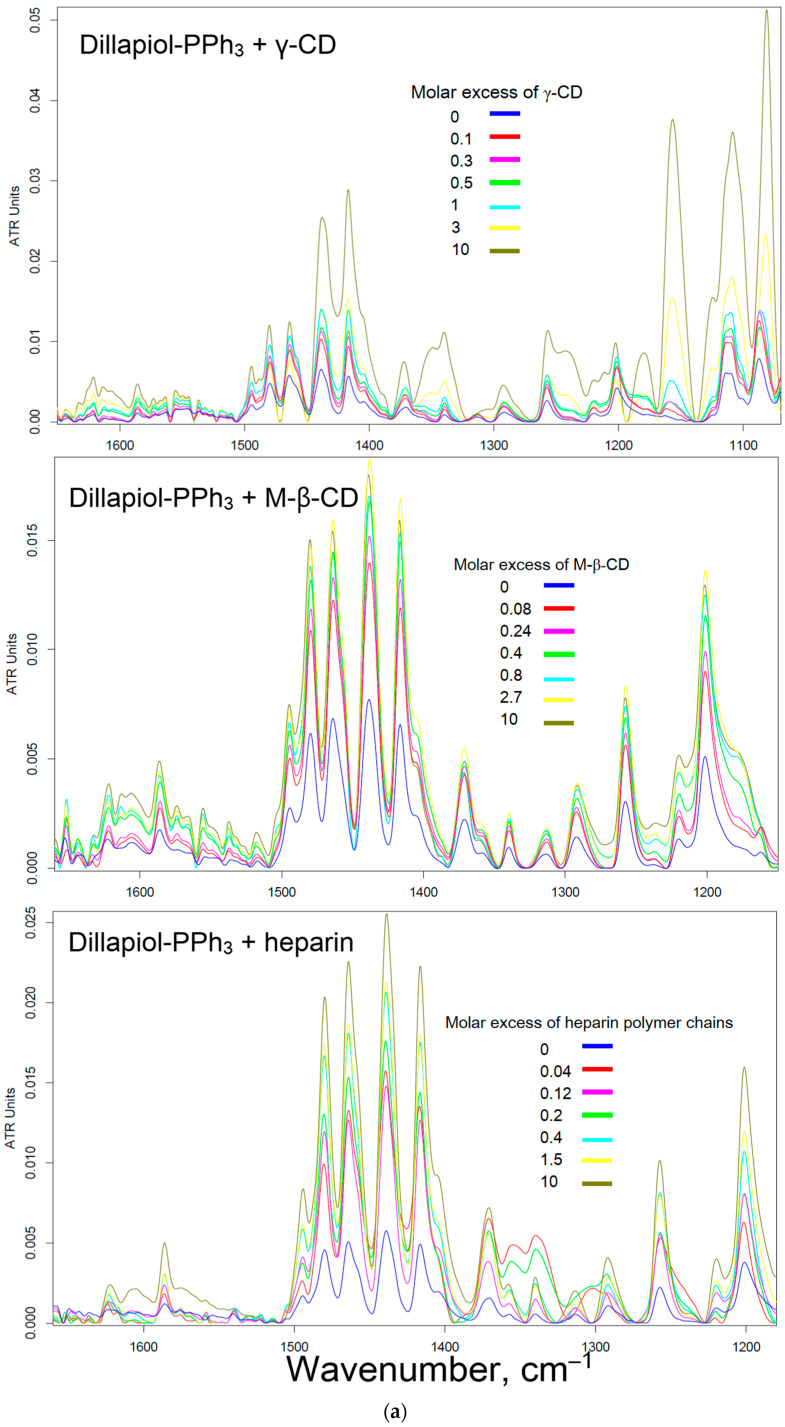

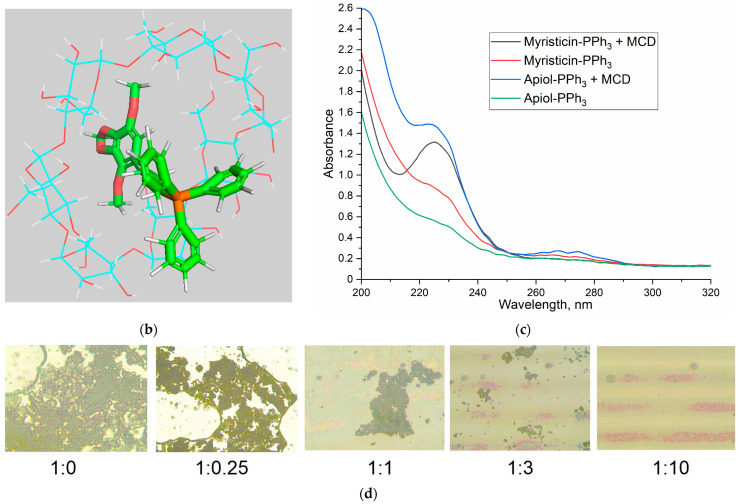
(**a**) FTIR spectra of dillapiol-PPh_3_ with γ-CD, M-β-CD and heparin. PBS (0.01 M, pH 7.4). T(incubation) = 40 °C. T(registration) = 22 °C. (**b**) The proposed structure of the β-cyclodextrin complex with apiol-PPh_3_ (for other compounds, the structure is similar). (**c**) UV spectra of myristicin-PPh_3_, apiol-PPh_3_ and the equimolar complexes with M-β-CD. (**d**) Micrographs of samples of apiol-PPh_3_ and its complexes with M-β-CD in the molar ratio from 1:0, 1:0.25, 1:1, 1:3 to 1:10.

FTIR spectroscopy provides valuable data on the interaction of molecules, including those applicable to characterize the non-covalent complexes of apiol-PPh_3_ (and analogues) with cyclodextrins and heparin. In the FTIR spectra of apiol-PPh_3_ and analogues (Figure 3a), one characteristic is the bands of valence oscillations of the bonds C=C of the aromatic system (1450–1650 cm^−1^) overlapping with the bands of deformation oscillations C-H (1400–1500 cm^−1^). The intensity of these peaks increases with the formation of non-covalent complexes of apiol-PPh_3_ and its analogues with cyclodextrins and heparin due to the transition of the solid phase into solution. Linear fitting of the intensity of peaks in the FTIR spectra on the cyclodextrin or heparin concentration (Section 2.3) in Hill coordinates allows determining the dissociation constants of the complexes (Table 2). The interactions of triphenylphosphine derivatives of allylbenzenes with γ-CD is rather weak (K_d_ 10 mM values). In the case of M-β-CD, the dissociation constants reach millimolar values, which is sufficient to obtain soluble forms of adjuvants (Table 2). Thus, the β-cyclodextrin derivatives are more suitable in terms of size for inclusion of the adjuvants studied. Heparin forms rather strong complexes due to multipoint electrostatic interactions: *K*_d_ 10^−3^–10^−4^ M per heparin monomeric unit or 10^−5^ M per heparin molecule. Comparing the values of the dissociation constants of alkylbenzenes and PPh_3_-derivatives complexes with cyclodextrins, we observed that these *K*_d_ values are close, which means that it is the allylbenzene-fragment (of apiol-PPh_3_) that plunges into the cyclodextrin cavity, and the triphenylphosphine radical looks outward (Figure 3b), which would provide the implementation of mitochondrial targeting of the developed formulations. 

Figure 3c shows the UV spectra of myristicin-PPh_3_, apiol-PPh_3_ and their complexes with M-β-CD: PPh_3_ derivatives due to their low solubility in water do not have a clearly defined spectrum. On the contrary, their complexes with M-β-CD are soluble, and a clear peak in the UV spectrum is pronounced (225 nm). Based on UV spectra data in the presence of M-β-CD, the increase in solubility of apiol-PPh3 and analogues by a second order of magnitude is observed (Table 2). 

Visually, the dissolution of PPh_3_ derivatives of allylbenzenes is observed in a light microscope (Figure 3d–g): with an increase in the molar excess of cyclodextrin, an increasing number of inclusion complexes are formed and, consequently, solubility increases (1:1 molar ratio) and crystals decrease to complete dissolution (10-fold molar excess of M-β-CD).

### 2.4. Anticancer Activity of PPh_3_ Derivatives and Formulations

**Figure 4:**
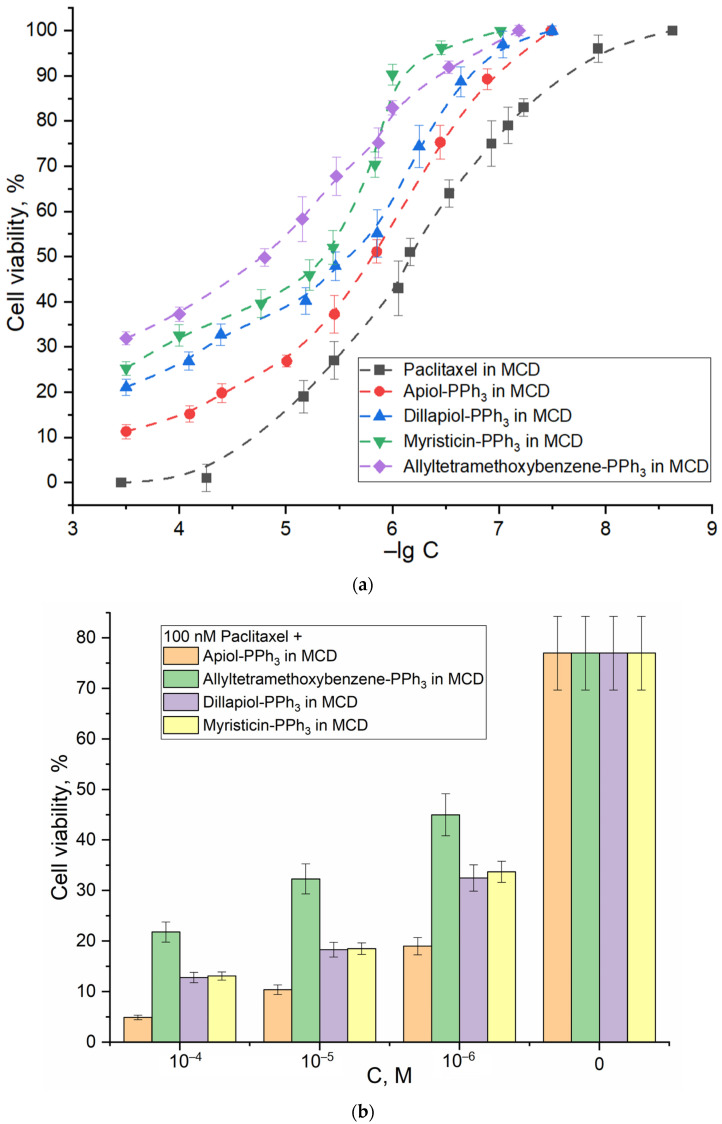
Viability of A549 cells in the presence of paclitaxel and allylbenzenes’ PPh_3_ derivatives in the form of complexes with M-β-CD (1:5 mol/mol). MTT assay. (**a**) Dose–effect graphs. (**b**) MTT assay results for 100 nM paclitaxel alone and combined with allylbenzenes’ PPh_3_ derivatives in the form of complexes with M-β-CD (1:5 mol/mol).

Previously, we demonstrated the antitumor activity of apiol, eugenol and their analogues from the allylbenzene class, and we showed the ability of these substances to act as efflux pump inhibitors and as a membrane-penetrating enhancer agent [49]. Apparently PPh_3_ derivatives effectively penetrate into cancer cells along a potential gradient, inhibit efflux proteins and mitochondrial enzymes, causing the apoptosis of cancer cells, with IC_50_ values being lower by two orders of magnitude than the corresponding allylbenzene (Table 3). Surprisingly, PPh_3_ derivatives (especially apiol-PPh_3_) in the complex with M-β-CD are close in terms of the cytostatic efficiency to the well-known cytotoxic drug paclitaxel (Figure 4a, Table 3); in addition, they demonstrate synergy with paclitaxel, with a synergy coefficient over 2 (indicating strong synergy). For allylbenzenes, a synergy close to additivity (the cytostatic effect of adjuvant + paclitaxel is almost equal to the sum of their individual contributions) was observed; for PPh_3_ derivatives, a pronounced increase in the action of paclitaxel is characteristic (the cytostatic effect of adjuvant + paclitaxel is much higher (>) than the sum of their individual contributions). For apiol-PPh_3_, the most pronounced effect of increasing the activity of paclitaxel was observed (Figure 4b).

The mechanism of synergistic action is that the main cytostatic and its adjuvant act on different targets. The PPh_3_ fragment serves as an intermediary for the delivery of allylbenzenes to the mitochondria of cancer cells, which is where the inhibition of enzymes (dehydrogenases) occurs. Paclitaxel disrupts the cycle of cell division. Meanwhile, CD improves the solubility of drugs and increases adsorption and absorption by cancer cells, which in total affects the increase in the effectiveness of the cytostatic drug. The potential of inclusion of antitumor drugs in CD in terms of enhancing penetration into cancer cells was demonstrated also in our previous work [49].

Thus, allylbenzene-PPh_3_ inclusion complexes with cyclodextrin are potentially applicable in medicine as antitumor drugs. At the same time, it is important to find out the selectivity of the cytostatic action of the formulation developed against cancer cells and the safety of these formulations for normal cells. 

### 2.5. Selectivity of Action and Safety of Cytotoxic Formulations Developed

#### 2.5.1. HEK293T as Normal Cell Model

HEK293T are model normal (non-cancer) cells that are widely used to compare the selectivity of cytostatic formulations on cancer cells [49]. Quantitative data on the safety and selectivity of action for the formulations based on PPh_3_ derivatives are presented in Table 4. According to the MTT test, the concentration of cytostatics of 100 µM causes the death of up to 85% of cancer cells A549 (Figure 4a), while for non-cancer cells (HEK293T), the dying rate is only 15–20%.

**Figure 5:**
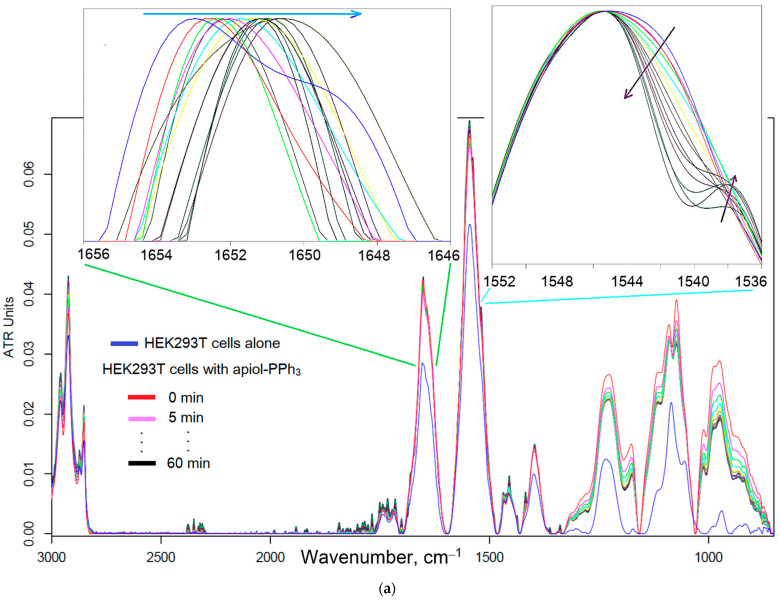

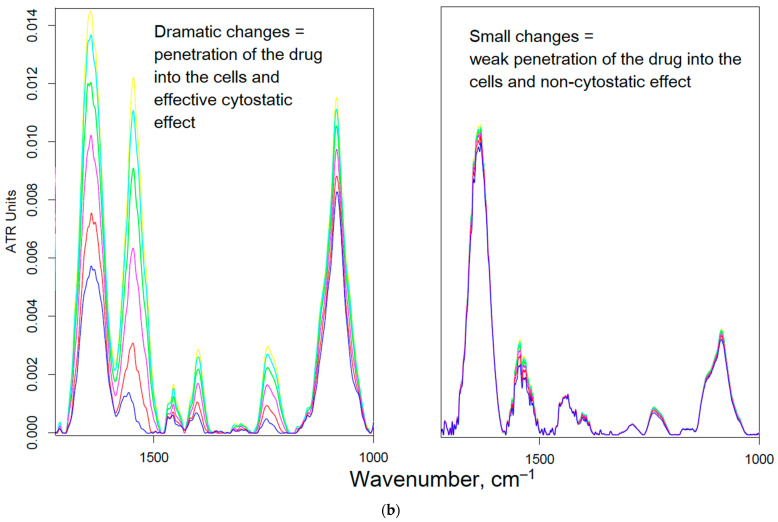
(**a**) FTIR spectra of HEK293T cells during online incubation (with step 5 min) with apiol-PPh_3_ in the form of inclusion complexes with M-β-CD. The inserts show enlarged fragments of peaks of amide I and II with a normalized intensity for the better visualization of shifts of maxima. T = 37 °C. The inserts show enlarged fragments of peaks of amide I and II with a normalized intensity for better visualization of shifts of maxima. (**b**) FTIR spectra of HEK293T cells pre-incubated with doxorubicin (**left**), doxorubicin in “intelligent” micelles [87] (**right**) as a control of the correlation of changes in the intensity of peaks with the penetration and cytostatic effect of the drug.

Earlier, we showed that the data of FTIR spectroscopy correlate with the data of the MTT test on cell survival [49,83]. The main cell structural units that contribute to the absorption of IR diapason can be assigned as follows (Figure 5): lipids of the cell membrane (2800–3000 cm^−1^), proteins, especially transmembrane (1500–1700 cm^−1^), phosphate groups of DNA (1240 cm^−1^) and carbohydrates, including lipopolysaccharides (900–1100 cm^−1^). Previously, we developed a technique for tracking the penetration and adsorption of the drug into cells using FTIR spectroscopy: dramatic changes in the intensity of the peaks of amide 1 and amide 2 indicate effective penetration of the drug into cells and vice versa [49,87,88].

Here, we present the real-time data of FTIR spectroscopy during the incubation of a suspension of HEK293T cells with apiol-PPh_3_ in M-β-CD (Figure 5). Comparing the red spectrum (at 0 min incubation) and the black spectrum (after 60 min), it is obvious that there are practically no changes in the intensity of the peaks of amide I and II, characterizing the interaction of the drug with trans-membrane proteins, and indicating drug penetration. There is only a shift of the peak of amide 1 to the low-frequency region (inserts in Figure 5, the normalized intensity is shown) and amide 2 to the high-frequency region with the simultaneous appearance of the shoulder. This indicates the only adsorption of drug molecules on the cell surface, which is also confirmed by a weak increase in the intensity of the peaks at 2850–3000 cm^−1^ corresponding to the valence vibrations of the CH_2_ groups (lipid bilayer). Thus, PPh_3_ derivatives show only marginal activity against normal cells. For comparison, we present a positive control of the active and inactive reagent on the HEK293T cells (Figure 5b). Dramatic changes in the intensity of the amide 1 and amide 2 peaks (Figure 5b, left) indicate the penetration of the model cells’ membrane-penetrating drug (doxorubicin) into the cells and effective cytostatic effect (according to MTT test). On the contrary, small changes in the intensity of the peaks of amide 1 and amide 2 indicate the weak penetration of the drug into the cells and the non-cytostatic effect of doxorubicin in the composition with “intelligent” micelles (Figure 5b, right).

#### 2.5.2. Hemolytic Activity, Thrombogenicity and Phenotypic Sea Urchin Embryo Assay

Hemolytic activity and thrombogenicity are the primary parameters for evaluating the safety of medical formulations [89,90,91]. The phenotypic sea urchin embryo assay developed by colleagues is a visual way to study the toxicity of formulations in vivo. The sea urchin and human genomes contain more than 7000 common genes, including orthologs associated with a number of human diseases. Therefore, sea urchins can be considered as a reliable and versatile model organism for studying the safety of new and existing cytotoxic formulations in vivo. Table 5 presents data on the % of erythrocyte hemolysis, the degree of whole blood thrombosis of apiol-PPh_3_ and analogues, and data of phenotypic sea urchin embryo assay. Thus, the non-toxicity of PPh_3_ derivatives of allylbenzenes for normal non-cancer cells, as well as the selectivity of action against cancer cells, is shown. The selectivity of cytotoxic action against cancer cells in comparison with normal cells can be explained by the fact that the PPh3- cation provides selective accumulation and reduction in the mitochondrial membrane potential of the transformed cancer cells [92]. Cyclodextrin (M-β-CD) and heparin are non-toxic (and approved by the FDA for intravenous application).

## 3. Materials and Methods

### 3.1. Reagents

γ-cyclodextrin (γ-CD) and methyl β-cyclodextrin (M-β-CD) were purchased from Sigma Aldrich (St. Louis, MI, USA). Apiol, dillapiol, allyltetramethoxybenzene and myristicin were isolated from plant extracts as described earlier [77]. Heparin (MM 50–80 kDa), organic solvents, salts and acids were from Reakhim (Moscow, Russia). 

The synthesis of triphenylphosphine derivatives of allylbenzenes was performed as described earlier in the work [46]. 

### 3.2. Characterization of PPh_3_ Adjuvants Using NMR Spectroscopy

The ^1^H and ^13^C NMR spectra of the apiol, apiol-PPh_3_ and analogues in d_6_-DMSO were recorded on a Bruker Avance 400 spectrometer (Bruker Biospin, Rheinstetten, Germany) at an operating frequency of 400 MHz. The chemical shifts are shown in ppm on the δ scale relative to hexamethyldisiloxane as an internal standard. The analysis and processing of the NMR spectra were performed with the program MestReNova v.12.0.0–20080).

### 3.3. Non-Covalent Complexes of Apiol, Apiol-PPh_3_ and Analogues with Cyclodextrins and Heparin, Preparation and Characteristics

Non-covalent complexes of apiol, apiol-PPh_3_ and analogues with cyclodextrins (with different molar ratios) and heparin (15 kDa, 1:1 *w*/*w*) were obtained by adding solutions of cyclodextrins (100 mg/mL) or heparin (20 mg/mL) in PBS to apiol or apiol-PPh_3_ (or analogues) samples (2–3 mg). The excess of CDs or heparin was varied from 0.04 to 10 mol/mol. Then, mixtures were incubated for 1 h at 40 °C. Cyclodextrin or heparin are necessary for the solubilization of extremely poorly soluble compounds.

Concentrations of the active substance varied from 10^−2^ to 10^−4^ M. For MTT assay and biological experiments, substances in the concentration range from 10^−3^ to 10^−9^ M were studied by the dilution of initial ones in a cell growth medium or buffer.

The solubility of apiol and apiol-PPh_3_ (or analogues) in aqueous solution was determined by UV spectroscopy. UV spectra were recorded on the UltraSpec 2100 pro device (AmerSham Biosciences, Cambridge, UK) three times. The substances were dissolved in acetonitrile followed by recording the UV spectra at various cytostatics’ concentrations, and then we plotted calibration dependencies. Next, saturated solutions of substances in water were prepared, and the spectra of the aqueous solutions were recorded. Considering the extinction coefficients in water and acetonitrile to be approximately equal, the solubility was determined.

### 3.4. MCD Inclusion Complexes Synthesis

The inclusion complexes of Paclitaxel with MCD were prepared as described in our previous paper [49].

### 3.5. Determination of the Dissociation Constants of Complexes of Apiol-PPh_3_ and Analogues with Cyclodextrins and Heparin Using FTIR Spectroscopy

The ATR-FTIR spectra of samples (Section 3.3) were acquired using a Bruker Tensor 27 spectrometer equipped with a liquid N_2_ cooled MCT (mercury cadmium telluride) detector. Samples were placed in a thermostatic cell BioATR-II with ZnSe ATR element (Bruker, Germany). FTIR spectra were recorded from 850 to 4000 cm^−1^ with 1 cm^−1^ spectral resolution; 50 scans were accumulated and averaged. Spectral data were processed using the Bruker software system Opus 8.2.28 (Bruker, Germany). The spectrum of cyclodextrin or heparin in the corresponding concentration was subtracted from the spectra of the complexes. Then, the dependencies of the peak intensities of the corresponding C=C oscillation (aromatic system of apiol-PPh_3_ and analogues (1475–1510 cm^−1^)) was constructed, which least overlaps with the spectrum of cyclodextrin and heparin.

Calculation of the dissociation constants X − M-β-CD, X − γ-CD and X − heparin, where X is Apiol-PPh_3_ and analogues, was performed as follows:

(1) Consider the equilibrium (given for the M-β-CD, for the rest, it is the same): X + nM-β-CD ↔ X · nM-β-CD, where *K*_d_ = [M-β-CD]^n^ · [X]/[X · nM-β-CD];
(2) Complexation degree calculation θ = (ξ − ξ_0_)/(ξ∞ − ξ_0_), where ξ is FTIR peak current intensity, ξ_0_ is FTIR peak initial intensity (only Apiol-PPh_3_ and analogues without M-β-CD, etc), ξ∞ is FTIR peak intensity of Apiol-PPh_3_ and analogues with a large excess of M-β-CD, etc.;
(3) Linear fitting of data: lg (θ/(1 − θ)) versus logarithm of concentration of the M-β-CD, γ-CD or heparin was carried out using the Hill equation: lg (θ/(1 − θ)) = n · lg [M-β-CD] − lg *K*_d_.

### 3.6. Confirmation of Particle Formation and Their Characterization 

Confirmation of particle formation was carried out using Atomic Force Microscopy (*NTEGRA II* Moscow, Russia), and Fourier Infrared Microscopy (Micran-3 IR microscope, Simex, Novosibirsk, Russia). 

Topography, phase and magnitude signal images of the micelles deposited onto freshly cleaved surface of mica were obtained by AFM microscopy using a scanning probe microscope NTEGRA operated in a semi-contact mode with 15–20 nm peak-to-peak amplitude of the “free air” probe oscillations. Using AFM, the formation of ordered particles of heparin complexes with drugs (100–150 nm) from disordered heparin aggregates (50–480 nm) was observed.

Using IR microscopy, dry powders of drugs and their complexes with CD and heparin were studied: they showed a uniform distribution of the drug over the area of the studied sample (i.e., evenly inclusion in molecular containers). 

Circular dichroism spectra of heparin were recorded on the Jasco J-815 CD Spectrometer (Tokyo, Japan) for the determination of heparin in the tested formulations. 

### 3.7. Cell Cultivation and Determination of Cytotoxic Activity

Adenocarcinomic human alveolar basal epithelial cells A549 cell lines (Manassas, VA, USA) were cultured in RPMI-1640 medium, linear cells of the embryonic kidney human epithelium (HEK293T) were grown in DMEM medium as described earlier [49]. Cell lines were obtained from Lomonosov Moscow State University Depository of Live Systems Collection and Laboratory of Medical Biotechnology, Institute of Biomedical Chemistry (Moscow, Russia).

The cytotoxic activity of paclitaxel, Apiol-PPh_3_ and analogues was determined using an MTT test [49]. Paclitaxel-adjuvant synergism coefficients (SC) were calculated as CV (paclitaxel) × CV (alone adjuvant)/CV (combo paclitaxel + adjuvant), where CV represents the cell viability. The synergy coefficient can be interpreted as strong synergy (SC > 2), synergy (2 > SC > 1.2), indifference/additivity (1.2 > SC > 0.8), antagonism (0.8 > SC > 0.5), and inhibition (SC < 0.5), as described earlier [49,77].

### 3.8. Phenotypic Sea Urchin Embryo Assay

Adult sea urchins, *Paracentrotus lividus* L. (Echinidae), were collected from the Mediterranean Sea on the Cyprus coast and kept in an aerated seawater tank and were used to study the cleavage alteration of Apiol-PPh_3_ and analogues [46,93]. Experiments with sea urchin embryos comply with the requirements of biological ethics. Artificial spawning does not lead to the death of animals, embryos develop outside the female body, and both adult sea urchins after spawning and an excess of intact embryos return to the sea, which is their natural habitat.

### 3.9. Study of the Safety of Formulations (Hemolytic Activity and Thrombogenicity)

The hemolytic activity and thrombogenicity of apiol-PPh_3_ and analogues were studied using an earlier published technique [83].

### 3.10. Statistical Analysis

Statistical analysis of cytotoxicity and spectral data was performed using Student’s *t*-test Origin 2022 software (OriginPro 2022 v.9.9.0.225, OriginLab Corporation, Northampton, MA, USA). Values are given as the mean ± SD of three or five experiments.

## 4. Conclusions

In this paper, soluble forms (inclusion complexes in cyclodextrins or complexes with polyanionic polymer) of triphenylphosphine derivatives of allylbenzenes (individual components of plant (parsley) essential oils) are presented as potential independent cytostatic drugs (IC_50_ are in the micromolar concentration range (10^−6^ M) against A549) and as adjuvants to the classical cytotoxic drug paclitaxel. The positively charged PPh_3_ fragment is used as an address label for the preferential delivery of apiol and its analogues to the mitochondria of cancer cells, which is possible due to altered metabolism in cancer cells. Allylbenzene-PPh_3_ enhances the effect of paclitaxel by 1.5–2 orders of magnitude in terms of IC_50_. At the same time, a high selectivity of the action of cytostatics against cancer cells is achieved and, practically, the drugs do not act on healthy HEK293T model cells. In addition, the high safety of triphenylphosphine formulations for erythrocytes, thrombosis and sea urchin embryos has been shown. In many cancers, the mitochondrial membrane is more prone to depolarization as compared to normal cells, which probably explains the observed selectivity of our compounds, since PPh_3_ derivatives are known to act as mitochondria-targeting agents. Furthermore, their efficacy as adjuvants may be the most pronounced in combination (or in conjugation) with anticancer drugs whose mechanism of action affects mitochondria or the mitochondrial membrane, which is an emerging field in cancer research. 

## Figures and Tables

**Table 1:** Positions of characteristic peaks in the FTIR spectra of dillapiol, allyltetramethoxybenzene, PPh_3_, dillapiol-PPh_3_, and allyltetramethoxyallylbenzene-PPh_3_.

Compound	Functional Group *	Position of the Characteristic Peak in the FTIR Spectra, cm^−1^	
		Octane–Ethanol (50:50 *v*:*v*)	Water–Ethanol (50:50 *v*:*v*)
Dillapiol	O–CH_2_–O	2917	2924
	=C–O–C	1065	1045
	–O–CH_3_	2848	2858
	C–C aromatic	1464	1448
Allyltetramethoxybenzene	Aryl–CH_2_–CH=CH_2_	2956	2930
	–O–CH_3_	2924	2901
	C–C aromatic	1492 and 1466	1488 and 1449–1456
Propyl-PPh_3_	C–C aromatic	1421	1414–1420
		1440 and 1455	1455
Dillapiol-PPh_3_	O–CH_2_–O	2937–2952	2927–2932 (2928)
	=C–O–C	1082–1087	1086 (1088)
	–O–CH_3_	2848	–
	Aryl–CH_2_–CH_2_–CH_2_–PPh_3_	2970	2981 (2974)
	C–C aromatic	1502	1485
		1455 and 1465	1448–1457
Allyltetramethoxybenzene-PPh_3_	=C–O–C	1086	1089 (1088)
	–O–CH_3_	2855	2900 (2880–2900)
	Aryl–CH_2_–CH_2_–CH_2_–PPh_3_	2993 and 2957	2980 (2974)
	C–C aromatic	1467	1482–1488 (1486)

* the atoms whose bond oscillations are observed are underlined.

**Table 2:** Dissociation constants of complexes of adjuvants and cyclodextrins or heparin. Solubility of X and X-PPh_3_ in PBS and solubility of their complexes with M-β-CD in PBS. Comparison of unmodified “apiols” and the PPh_3_ derivatives.

Substance X-PPh_3_	−lg *K*_d_ (X − M-β-CD) *	−lg *K*_d_ (X − γ-CD) **	−lg *K*_d_ (X − Heparin) ***	Solubility in PBS, mM	Solubility in the Presence of 0.05 M M-β-CD, mM
Apiol-PPh_3_	2.9 ± 0.3	1.2 ± 0.2	2.7 ± 0.2	0.08 ± 0.01	15 ± 2
Dillapiol-PPh_3_	2.6 ± 0.2	1.4 ± 0.3	3.0 ± 0.3	0.09 ± 0.01	8 ± 1
Myristicin-PPh_3_	3.0 ± 0.3	1.3 ± 0.1	2.6 ± 0.2	0.04 ± 0.005	12 ± 3
Allyltetramethoxybenzene-PPh_3_	3.1 ± 0.2	2.1 ± 0.2	3.2 ± 0.1	0.07± 0.01	17 ± 5
Substance X	−lg *K*_d_ (X − M-β-CD) ****			Solubility in PBS, mM	Solubility in the presence of 0.05 M M-β-CD, mM
Apiol	2.6 ± 0.3			0.13 ± 0.01	22 ± 4
Dillapiol	2.7 ± 0.5			0.24 ± 0.05	27 ± 3
Myristicin	3.5 ± 0.2			0.030 ± 0.007	41 ± 5
Allyltetramethoxybenzene	3.4 ± 0.3			0.16 ± 0.02	38 ± 2

* The complex with M-β-CD is formed in a molar ratio of 1 to 1; ** The complex with γ-CD is formed in molar excess of **X** approximately 1.2–1.4; *** Dissociation constants were calculated per one unit of heparin by the formula C_12_H_19_NO_20_S_3_; ****Data from paper [77].

**Table 3:** Anti-A549 activity of allylbenzenes, its PPh_3_ derivatives: alone (middle column) and combined with paclitaxel in M-β-CD (right column).

Substance X in M-β-CD	−lg (IC50) of X in M-β-CD * against A549	Synergy Coefficients of Adjuvants and PPh_3_–Adjuvants with Paclitaxel **
Paclitaxel	6.2 ± 0.2	-
Apiol-PPh_3_	5.8 ± 0.1	2.2 ± 0.2
Dillapiol-PPh_3_	5.6 ± 0.2	1.5 ± 0.1
Myristicin-PPh_3_	5.3 ± 0.2	1.8 ± 0.3
Allyltetramethoxybenzene-PPh_3_	4.8 ± 0.1	1.3 ± 0.1
Apiol	3.6 ± 0.3	1.3 ± 0.2
Dillapiol	3.2 ± 0.1	1.1 ± 0.1
Myristicin	2.9 ± 0.3	0.9 ± 0.2
Allyltetramethoxybenzene	3.5 ± 0.2	1.4 ± 0.2

* 1:5 mol/mol; ** X − M-β-CD was studied. Synergy coefficient (SC) can be interpreted as strong synergy (SC > 2), synergy (2 > SC > 1.2), indifference/additivity (1.2 > SC > 0.8), antagonism (0.8 > SC > 0.5), inhibition (SC < 0.5). For all the studied compounds, the difference between the cytostatic effect of a combination of two substances is statistically significantly different from the effects of single substances: *p* < 0.01.

**Table 4:** Anti-HEK293T activity of PPh_3_ derivatives of allylbenzenes in M-β-CD as a criterion for the safety of medicinal formulations. MTT assay. RPMI-1640 medium supplemented with 5% fetal bovine serum and 1% sodium pyruvate at 5% CO_2_/95% air in a humidified atmosphere at 37 °C.

Substance X in M-β-CD	HEK293T Viability (%) at C_X_ = 300 µM	HEK293T Viability (%) at C_X_ = 100 µM	HEK293T Viability (%) at C_X_ = 10 µM
Apiol-PPh_3_	71 ± 2	82 ± 3	93 ± 2
Dillapiol-PPh_3_	70 ± 5	84 ± 5	95 ± 3
Myristicin-PPh_3_	75 ± 3	91 ± 2	97 ± 3
Allyltetramethoxybenzene-PPh_3_	83 ± 4	88 ± 3	98 ± 1

**Table 5:** Safety data on triphenylphosphine derivatives and paclitaxel in the complex with M-β-CD.

Substance X in M-β-CD	Hemolysis Index *, %	Thrombosis Index **, %	Concentration Causing Changes in Sea Urchin Embryos, μM
Paclitaxel	<0.5 (*p* = 0.012)	0.6 ± 0.1	>4 ***
Apiol-PPh_3_	0.8 ± 0.2	1.1 ± 0.2	
Dillapiol-PPh_3_	0.9 ± 0.2	1.0 ± 0.1	
Myristicin-PPh_3_	0.5 ± 0.1	1.5 ± 0.2	
Allyltetramethoxybenzene-PPh_3_	0.7 ± 0.1	0.7 ± 0.2	

* For 0.1 mg/mL samples. The amount of released hemoglobin from erythrocytes relative to the control sample containing 0.05% Triton X-100; ** For 0.1 mg/mL samples. The amount of non-released hemoglobin in thrombus when H_2_O was added relative to the control sample containing microscopic glass particles; *** *p* < 0.05.

## Data Availability

The data presented in this study are available in the main text and Supplementary Materials.

## Supplementary Materials

The following supporting information can be downloaded at: https://www.mdpi.com/article/10.3390/ph16121651/s1, Figure S1. ^1^H NMR spectra of (a) dillapiol, (b) dillapiol-PPh_3_, (c) myristicin, (d) myristicin-PPh_3_. T = 25 °C. d_6_-DMSO. 400 MHz.

## Abbreviations

γ-CD	γ-cyclodextrin
M-β-CD	methyl β-cyclodextrin

## References

[1] Malebari A.M., Wang S., Greene T.F., O’Boyle N.M., Fayne D., Khan M.F., Nathwani S.M., Twamley B., McCabe T., Zisterer D.M. (2021). Synthesis and antiproliferative evaluation of 3-chloroazetidin-2-ones with antimitotic activity: Heterocyclic bridged analogues of combretastatin a-4. Pharmaceuticals.

[2] Muniz D.F., dos Santos Barbosa C.R., de Menezes I.R.A., de Sousa E.O., Pereira R.L.S., Júnior J.T.C., Pereira P.S., de Matos Y.M.L.S., da Costa R.H.S., de Morais Oliveira-Tintino C.D. (2021). In vitro and in silico inhibitory effects of synthetic and natural eugenol derivatives against the NorA efflux pump in Staphylococcus aureus. Food Chem..

[3] Demchuk D.V., Samet A.V., Chernysheva N.B., Ushkarov V.I., Stashina G.A., Konyushkin L.D., Raihstat M.M., Firgang S.I., Philchenkov A.A., Zavelevich M.P. (2014). Synthesis and antiproliferative activity of conformationally restricted 1,2,3-triazole analogues of combretastatins in the sea urchin embryo model and against human cancer cell lines. Bioorganic Med. Chem..

[4] Cox G., Wright G.D. (2013). Intrinsic antibiotic resistance: Mechanisms, origins, challenges and solutions. Int. J. Med. Microbiol..

[5] Alqahtani F.Y., Aleanizy F.S., El Tahir E., Alkahtani H.M., AlQuadeib B.T. (2019). Paclitaxel. Profiles Drug Subst. Excip. Relat. Methodol..

[6] Iyer A.K.V. (2018). Ionophores: Potential Use as Anticancer Drugs and Chemosensitizers. Cancers.

[7] Frieri M., Kumar K., Boutin A. (2017). Antibiotic resistance. J. Infect. Public Health.

[8] Dasari S., Bernard Tchounwou P. (2014). Cisplatin in cancer therapy: Molecular mechanisms of action. Eur. J. Pharmacol..

[9] Mancuso G., Midiri A., Gerace E., Biondo C. (2021). Bacterial antibiotic resistance: The most critical pathogens. Pathogens.

[10] Larsson M., Huang W.C., Hsiao M.H., Wang Y.J., Nydén M., Chiou S.H., Liu D.M. (2013). Biomedical applications and colloidal properties of amphiphilically modified chitosan hybrids. Prog. Polym. Sci..

[11] Macêdo N.S., Silveira Z.D.S., Patrícia P., Cordeiro M., Douglas H., Coutinho M., Pinto J., Júnior S., José L., Júnior Q. (2022). Inhibition of Staphylococcus aureus Efflux Pump by O-Eugenol and Its Toxicity in Drosophila melanogaster Animal Model. BioMed Res. Int..

[12] Seneme E.F., dos Santos D.C., de Lima C.A., Zelioli Í.A.M., Sciani J.M., Longato G.B. (2022). Effects of Myristicin in Association with Chemotherapies on the Reversal of the Multidrug Resistance (MDR) Mechanism in Cancer. Pharmaceuticals.

[13] Sritharan S., Sivalingam N. (2021). A comprehensive review on time-tested anticancer drug doxorubicin. Life Sci..

[14] Junnuthula V., Kolimi P., Nyavanandi D., Sampathi S., Vora L.K., Dyawanapelly S. (2022). Polymeric Micelles for Breast Cancer Therapy: Recent Updates, Clinical Translation and Regulatory Considerations. Pharmaceutics.

[15] Syed S.B., Lin S.Y., Arya H., Fu I.H., Yeh T.K., Charles M.R.C., Periyasamy L., Hsieh H.P., Coumar M.S. (2021). Overcoming vincristine resistance in cancer: Computational design and discovery of piperine-inspired P-glycoprotein inhibitors. Chem. Biol. Drug Des..

[16] Ishikawa T., Wright C.D., Ishizuka H. (1994). GS-X pump is functionally overexpressed in cis- diamminedichloroplatinum(II)-resistant human leukemia HL-60 cells and down-regulated by cell differentiation. J. Biol. Chem..

[17] Cho K., Wang X., Nie S., Chen Z., Shin D.M. (2008). Therapeutic nanoparticles for drug delivery in cancer. Clin. Cancer Res..

[18] Taghizadeh B., Taranejoo S., Monemian S.A., Moghaddam Z.S., Daliri K., Derakhshankhah H., Derakhshani Z. (2015). Classification of stimuli-responsive polymers as anticancer drug delivery systems. Drug Deliv..

[19] Zlotnikov I.D., Ezhov A.A., Petrov R.A., Vigovskiy M.A., Grigorieva O.A., Belogurova N.G., Kudryashova E.V. (2022). Mannosylated Polymeric Ligands for Targeted Delivery of Antibacterials and Their Adjuvants to Macrophages for the Enhancement of the Drug Efficiency. Pharmaceuticals.

[20] Zheng H., He W., Jiao W., Xia H., Sun L., Wang S., Xiao J., Ou X., Zhao Y., Shen A. (2021). Molecular characterization of multidrug-resistant tuberculosis against levofloxacin, moxifloxacin, bedaquiline, linezolid, clofazimine, and delamanid in southwest of China. BMC Infect. Dis..

[21] Karaiskos I., Lagou S., Pontikis K., Rapti V., Poulakou G. (2019). The “Old” and the “New” antibiotics for MDR Gram-negative pathogens: For whom, when, and how. Front. Public. Health.

[22] Marquez B. (2005). Bacterial efflux systems and efflux pumps inhibitors. Biochimie.

[23] Locci E., Lai S., Piras A., Marongiu B., Lai A. (2004). 13C-CPMAS and 1H-NMR study of the inclusion complexes of β-cyclodextrin with carvacrol, thymol, and eugenol prepared in supercritical carbon dioxide. Chem. Biodivers..

[24] Ulanowska M., Olas B. (2021). Biological properties and prospects for the application of eugenol—A review. Int. J. Mol. Sci..

[25] Weisheimer V., Miron D., Silva C.B., Guterres S.S., Schapoval E.E.S. (2010). Microparticles containing lemongrass volatile oil: Preparation, characterization and thermal stability. Pharmazie.

[26] Tadtong S., Watthanachaiyingcharoen R., Kamkaen N. (2014). Antimicrobial constituents and synergism effect of the essential oils from Cymbopogon citratus and Alpinia galanga. Nat. Prod. Commun..

[27] Singh G., Maurya S., deLampasona M.P., Catalan C.A.N. (2007). A comparison of chemical, antioxidant and antimicrobial studies of cinnamon leaf and bark volatile oils, oleoresins and their constituents. Food Chem. Toxicol..

[28] Yoo C.B., Han K.T., Cho K.S., Ha J., Park H.J., Nam J.H., Kil U.H., Lee K.T. (2005). Eugenol isolated from the essential oil of Eugenia caryophyllata induces a reactive oxygen species-mediated apoptosis in HL-60 human promyelocytic leukemia cells. Cancer Lett..

[29] Schepetkin I.A., Kushnarenko S.V., Özek G., Kirpotina L.N., Sinharoy P., Utegenova G.A., Abidkulova K.T., Özek T., Başer K.H.C., Kovrizhina A.R. (2016). Modulation of Human Neutrophil Responses by the Essential Oils from Ferula akitschkensis and Their Constituents. J. Agric. Food Chem..

[30] Teles A.M., Silva-Silva J.V., Fernandes J.M.P., Abreu-Silva A.L., Calabrese K.D.S., Mendes Filho N.E., Mouchrek A.N., Almeida-Souza F. (2021). GC-MS Characterization of Antibacterial, Antioxidant, and Antitrypanosomal Activity of Syzygium aromaticum Essential Oil and Eugenol. Evid.-Based Complement. Altern. Med..

[31] Hill L.E., Gomes C., Taylor T.M. (2013). Characterization of beta-cyclodextrin inclusion complexes containing essential oils (trans-cinnamaldehyde, eugenol, cinnamon bark, and clove bud extracts) for antimicrobial delivery applications. LWT-Food Sci. Technol..

[32] Zhang G., Yuan C., Sun Y. (2018). Effect of Selective Encapsulation of Hydroxypropyl-β-cyclodextrin on Components and Antibacterial Properties of Star Anise Essential Oil. Molecules.

[33] Herman A., Tambor K., Herman A. (2016). Linalool Affects the Antimicrobial Efficacy of Essential Oils. Curr. Microbiol..

[34] Abarca R.L., Rodríguez F.J., Guarda A., Galotto M.J., Bruna J.E. (2016). Characterization of beta-cyclodextrin inclusion complexes containing an essential oil component. Food Chem..

[35] Sadowska U., Matwijczuk A., Dródz T., Zabinski A., Niemczynowicz A. (2019). Spectroscopic Examination and Chemometric Analysis of Essential Oils Obtained from Peppermint. Processes.

[36] Valdivieso-Ugarte M., Gomez-Llorente C., Plaza-Díaz J., Gil Á. (2019). Antimicrobial, antioxidant, and immunomodulatory properties of essential oils: A systematic review. Nutrients.

[37] Boire N.A., Riedel S., Parrish N.M. (2013). Essential Oils and Future Antibiotics: New Weapons against Emerging’Superbugs’?. J. Anc. Dis. Prev. Rem..

[38] Dawidowicz A.L., Olszowy M. (2014). Does antioxidant properties of the main component of essential oil reflect its antioxidant properties? The comparison of antioxidant properties of essential oils and their main components. Nat. Prod. Res..

[39] Taylan O., Cebi N., Sagdic O. (2021). Rapid screening of mentha spicata essential oil and l-menthol in mentha piperita essential oil by atr-ftir spectroscopy coupled with multivariate analyses. Foods.

[40] Cardoso N.N.R., Alviano C.S., Blank A.F., Romanos M.T.V., Fonseca B.B., Rozental S., Rodrigues I.A., Alviano D.S. (2016). Synergism Effect of the Essential Oil from Ocimum basilicum var. Maria Bonita and Its Major Components with Fluconazole and Its Influence on Ergosterol Biosynthesis. Evid.-Based Complement. Altern. Med..

[41] Agatonovic-Kustrin S., Ristivojevic P., Gegechkori V., Litvinova T.M., Morton D.W. (2020). Essential oil quality and purity evaluation via ft-ir spectroscopy and pattern recognition techniques. Appl. Sci..

[42] Samet A.V., Shevchenko O.G., Rusak V.V., Chartov E.M., Myshlyavtsev A.B., Rusanov D.A., Semenova M.N., Semenov V.V. (2019). Antioxidant Activity of Natural Allylpolyalkoxybenzene Plant Essential Oil Constituents. J. Nat. Prod..

[43] Arana-Sánchez A., Estarrón-Espinosa M., Obledo-Vázquez E.N., Padilla-Camberos E., Silva-Vázquez R., Lugo-Cervantes E. (2010). Antimicrobial and antioxidant activities of Mexican oregano essential oils (Lippia graveolens H. B. K.) with different composition when microencapsulated inβ-cyclodextrin. Lett. Appl. Microbiol..

[44] Zlotnikov I.D., Davydova M.P., Danilov M.R., Krylov S.S., Belogurova N.G. (2023). Covalent Conjugates of Allylbenzenes and Terpenoids as Antibiotics Enhancers with the Function of Prolonged Action. Pharmaceuticals.

[45] Tsyganov D.V., Yakubov A.P., Konyushkin L.D., Firgang S.I., Semenov V.V. (2007). Polyalkoxybenzenes from plant sources 2. Synthesis of isoxazoline analogs of combretastatin from natural allyl(methylenedioxy)methoxybenzenes. Russ. Chem. Bull..

[46] Tsyganov D.V., Samet A.V., Silyanova E.A., Ushkarov V.I., Varakutin A.E., Chernysheva N.B., Chuprov-Netochin R.N., Khomutov A.A., Volkova A.S., Leonov S.V. (2022). Synthesis and Antiproliferative Activity of Triphenylphosphonium Derivatives of Natural Allylpolyalkoxybenzenes. ACS Omega.

[47] Semenov V.V., Rusak V.V., Chartov E.M., Zaretskii M.I., Konyushkin L.D., Firgang S.I., Chizhov A.O., Elkin V.V., Latin N.N., Bonashek V.M. (2007). Polyalkoxybenzenes from plant raw materials 1. Isolation of polyalkoxybenzenes from CO2 extracts of Umbelliferae plant seeds. Russ. Chem. Bull..

[48] Neuhaus-Carlisle K., Vierling W., Wagner H. (1997). Screening of plant extracts and plant constituents for calcium-channel blocking activity. Phytomedicine.

[49] Zlotnikov I.D., Dobryakova N.V., Ezhov A.A., Kudryashova E.V. (2023). Achievement of the selectivity of cytotoxic agents against cancer cells by creation of combined formulation with terpenoid adjuvants as prospects to overcome multidrug resistance. Int. J. Mol. Sci..

[50] Kamatou G.P.P., Vermaak I., Viljoen A.M., Lawrence B.M. (2013). Phytochemistry Menthol: A simple monoterpene with remarkable biological properties. Phytochemistry.

[51] Russin W.A., Hoesly J.D., Elson C.E., Tanner M.A., Gould M.N. (1989). Inhibition of rat mammary carcinogenesis by monoterpenoids. Carcinogenesis.

[52] Pereira de Lira M.H., Fernandes Queiroga Moraes G., Macena Santos G., Patrício de Andrade Júnior F., De Oliveira Pereira F., Oliveira Lima I. (2020). Synergistic antibacterial activity of monoterpenes in combination with conventional antimicrobials against Gram-positive and Gram-negative bacteria. Rev. Ciênc. Méd. Biol..

[53] Panche A.N., Diwan A.D., Chandra S.R. (2016). Flavonoids: An overview. J. Nutr. Sci..

[54] Ilyasov I.R., Beloborodov V.L., Selivanova I.A., Terekhov R.P. (2020). ABTS/PP decolorization assay of antioxidant capacity reaction pathways. Int. J. Mol. Sci..

[55] Jiso A., Khemawoot P., Techapichetvanich P., Soopairin S., Phoemsap K., Damrongsakul P., Wongwiwatthananukit S., Vivithanaporn P. (2022). Drug-Herb Interactions among Thai Herbs and Anticancer Drugs: A Scoping Review. Pharmaceuticals.

[56] Vaupel P., Kallinowski F., Okunieff P. (1989). Blood Flow, Oxygen and Nutrient Supply, and Metabolic Microenvironment of Human Tumors: A Review. Cancer Res..

[57] Williams A.G., Coleman G.S. (1997). The Rumen Microbial Ecosystem.

[58] Zlotnikov I.D., Kudryashova E.V. (2022). Spectroscopy Approach for Highly—Efficient Screening of Lectin—Ligand Interactions in Application for Mannose Receptor and Molecular Containers for Antibacterial Drugs. Pharmaceuticals.

[59] MacGowan A., Macnaughton E. (2017). Antibiotic resistance. Medicine.

[60] Ghezzi M., Pescina S., Padula C., Santi P., Del Favero E., Cantù L., Nicoli S. (2021). Polymeric micelles in drug delivery: An insight of the techniques for their characterization and assessment in biorelevant conditions. J. Control. Release.

[61] Garg A., Gupta B., Prakash R., Singh S. (2010). Preparation and characterization of hydroxypropyl-β-cyclodextrin inclusion complex of eugenol: Differential pulse voltammetry and 1H-NMR. Chem. Pharm. Bull..

[62] Cortés-Rojas D.F., Souza C.R.F., Oliveira W.P. (2014). Encapsulation of eugenol rich clove extract in solid lipid carriers. J. Food Eng..

[63] Akshaya R., Anjali A.K. (2021). Eugenol as Potential Medicine—Review. Ann. Rom. Soc. Cell Biol..

[64] Zari A.T., Zari T.A., Hakeem K.R. (2021). Anticancer Properties of Eugenol: A Review. Molecules.

[65] Pralhad T., Rajendrakumar K. (2004). Study of freeze-dried quercetin-cyclodextrin binary systems by DSC, FT-IR, X-ray diffraction and SEM analysis. J. Pharm. Biomed. Anal..

[66] Kayaci F., Ertas Y., Uyar T. (2013). Enhanced thermal stability of eugenol by cyclodextrin inclusion complex encapsulated in electrospun polymeric nanofibers. J. Agric. Food Chem..

[67] Gong L., Li T., Chen F., Duan X., Yuan Y., Zhang D., Jiang Y. (2016). An inclusion complex of eugenol into β-cyclodextrin: Preparation, and physicochemical and antifungal characterization. Food Chem..

[68] Seo S.J., Kim S.H., Sasagawa T., Choi Y.J., Akaike T., Cho C.S. (2004). Delivery of all trans -retinoic acid (RA) to hepatocyte cell line from RA / galactosyl a -cyclodextrin inclusion complex. Eur. J. Pharm. Biopharm..

[69] Fernandes C.M., Carvalho R.A., Pereira da Costa S., Veiga F.J.B. (2003). Multimodal molecular encapsulation of nicardipine hydrochloride by β-cyclodextrin, hydroxypropyl-β-cyclodextrin and triacetyl-β-cyclodextrin in solution. Structural studies by 1H NMR and ROESY experiments. Eur. J. Pharm. Sci..

[70] Brewster M.E., Loftsson T. (2007). Cyclodextrins as pharmaceutical solubilizers. Adv. Drug Deliv. Rev..

[71] Connors K.A. (1997). The stability of cyclodextrin complexes in solution. Chem. Rev..

[72] Sampaio C., Moriwaki C., Claudia A., Sato F., Luciano M., Medina A., Matioli G. (2014). Curcumin—B-cyclodextrin inclusion complex: Stability, solubility, characterisation by FT-IR, FT-Raman, X-ray diffraction and photoacoustic spectroscopy, and food application. Food Chem..

[73] Schneider H.J., Hacket F., Rüdiger V., Ikeda H. (1998). NMR studies of cyclodextrins and cyclodextrin complexes. Chem. Rev..

[74] Haimhoffer Á., Rusznyák Á., Réti-Nagy K., Vasvári G., Váradi J., Vecsernyés M., Bácskay I., Fehér P., Ujhelyi Z., Fenyvesi F. (2019). Cyclodextrins in drug delivery systems and their effects on biological barriers. Sci. Pharm..

[75] Cavalli R., Trotta F., Tumiatti W. (2006). Cyclodextrin-based nanosponges for drug delivery. J. Incl. Phenom. Macrocycl. Chem..

[76] Del Valle E.M.M. (2004). Cyclodextrins and their uses: A review. Process Biochem..

[77] Zlotnikov I.D., Belogurova N.G., Krylov S.S., Semenova M.N., Semenov V.V., Kudryashova E.V. (2022). Plant Alkylbenzenes and Terpenoids in the Form of Cyclodextrin Inclusion Complexes as Antibacterial Agents and Levofloxacin Synergists. Pharmaceuticals.

[78] Semenov V.V., Kiselyov A.S., Titov I.Y., Sagamanova I.K., Ikizalp N.N., Chernysheva N.B., Tsyganov D.V., Konyushkin L.D., Firgang S.I., Semenov R.V. (2010). Synthesis of antimitotic polyalkoxyphenyl derivatives of combretastatin using plant allylpolyalkoxybenzenes (1). J. Nat. Prod..

[79] Zlotnikov I.D., Vigovskiy M.A., Davydova M.P., Danilov M.R., Dyachkova U.D., Grigorieva O.A., Kudryashova E.V. (2022). Mannosylated Systems for Targeted Delivery of Antibacterial Drugs to Activated Macrophages. Int. J. Mol. Sci..

[80] Chernysheva N.B., Tsyganov D.V., Philchenkov A.A., Zavelevich M.P., Kiselyov A.S., Semenov R.V., Semenova M.N., Semenov V.V. (2012). Synthesis and comparative evaluation of 4-oxa- and 4-aza-podophyllotoxins as antiproliferative microtubule destabilizing agents. Bioorganic Med. Chem. Lett..

[81] Chudin A.A., Kudryashova E.V. (2022). Improved Enzymatic Assay and Inhibition Analysis of Redox Membranotropic Enzymes, AtGALDH and TcGAL, Using a Reversed Micellar System. Analytica.

[82] Chudin A.A., Zlotnikov I.D., Krylov S.S., Semenov V.V., Kudryashova E.V. (2023). Allylpolyalkoxybenzene Inhibitors of Galactonolactone Oxidase from Trypanosoma cruzi. Biochemistry.

[83] Zlotnikov I.D., Ezhov A.A., Ferberg A.S., Krylov S.S., Semenova M.N., Semenov V.V., Kudryashova E.V. (2023). Polymeric Micelles Formulation of Combretastatin Derivatives with Enhanced Solubility, Cytostatic Activity and Selectivity against Cancer Cells. Pharmaceuticals.

[84] Ghosh P., Vidal C., Dey S., Zhang L. (2020). Mitochondria targeting as an effective strategy for cancer therapy. Int. J. Mol. Sci..

[85] Wallace D.C. (2012). Mitochondria and cancer Douglas. Nat. Rev. Cancer.

[86] Zong W.X., Rabinowitz J.D., White E. (2016). Mitochondria and Cancer. Mol. Cell.

[87] Zlotnikov I.D., Streltsov D.A., Ezhov A.A. (2023). Smart pH- and Temperature-Sensitive Micelles Based on Chitosan Grafted with Fatty Acids to Increase the Efficiency and Selectivity of Doxorubicin and Its Adjuvant Regarding the Tumor Cells. Pharmaceuticals.

[88] Zlotnikov I.D., Ezhov A.A., Vigovskiy M.A., Grigorieva O.A., Dyachkova U.D., Belogurova N.G., Kudryashova E.V. (2023). Application Prospects of FTIR Spectroscopy and CLSM to Monitor the Drugs Interaction with Bacteria Cells Localized in Macrophages for Diagnosis and Treatment Control of Respiratory Diseases. Diagnostics.

[89] Gheran C.V., Voicu S.N., Galateanu B., Callewaert M., Moreau J., Cadiou C., Chuburu F., Dinischiotu A. (2022). In Vitro Studies Regarding the Safety of Chitosan and Hyaluronic Acid-Based Nanohydrogels Containing Contrast Agents for Magnetic Resonance Imaging. Int. J. Mol. Sci..

[90] Plenagl N., Duse L., Seitz B.S., Goergen N., Pinnapireddy S.R., Jedelska J., Brüßler J., Bakowsky U. (2019). Photodynamic therapy–hypericin tetraether liposome conjugates and their antitumor and antiangiogenic activity. Drug Deliv..

[91] Elshafie H.S., Sakr S.H., Sadeek S.A., Camele I. (2019). Biological Investigations and Spectroscopic Studies of New Moxifloxacin/Glycine-Metal Complexes. Chem. Biodivers..

[92] Frantsiyants E.M., Neskubina I.V., Sheiko E.A. (2020). Mitochondria of transformed cell as a target of antitumor influence. Res. Pract. Med. J..

[93] Semenova M.N., Demchuk D.V., Tsyganov D.V., Chernysheva N.B., Samet A.V., Silyanova E.A., Kislyi V.P., Maksimenko A.S., Varakutin A.E., Konyushkin L.D. (2018). Sea Urchin Embryo Model As a Reliable in Vivo Phenotypic Screen to Characterize Selective Antimitotic Molecules. Comparative evaluation of Combretapyrazoles, -isoxazoles, -1,2,3-triazoles, and -pyrroles as Tubulin-Binding Agents. ACS Comb. Sci..

