# Supplementary material for: Triphenylphosphine Derivatives of Allylbenzenes Express Antitumor and Adjuvant Activity When Solubilized with Cyclodextrin-Based Formulations
Source: Pharmaceuticals (Basel). 2023 Nov 26;16(12):1651. doi: 10.3390/ph16121651 (PMC10747112; doi:10.3390/ph16121651)
Supplement: Supplementary file 1 [file pharmaceuticals-16-01651-s001.zip › pharmaceuticals-2716345-supplementary.pdf]

Triphenylphosphine Derivatives of Allylbenzenes Express Antitumor and Adjuvant Activity When Solubilized with Cyclodextrin-Based Formulations

Igor D. Zlotnikov [1], Sergey S. Krylov [2], Marina N. Semenova [3], Victor V. Semenov [2] and Elena V. Kudryashova [1,*]

1 Faculty of Chemistry, Lomonosov Moscow State University, Leninskie Gory, 1/3, 119991 Moscow, Russia; zlotnikovid@my.msu.ru
2 N. D. Zelinsky Institute of Organic Chemistry RAS, 47 Leninsky Prospect, 119991 Moscow, Russia
3 N. K. Koltzov Institute of Developmental Biology RAS, 26 Vavilov Street, 119334 Moscow, Russia
* Correspondence: helenakoudriachova@yandex.ru

Figure S1. ^{1}H NMR spectra of (a) dillapiol, (b) dillapiol-PPh_3, (c) myristicin, (d) myristicin-PPh_3. T = 25 °C. d_6-DMSO. 400 MHz.

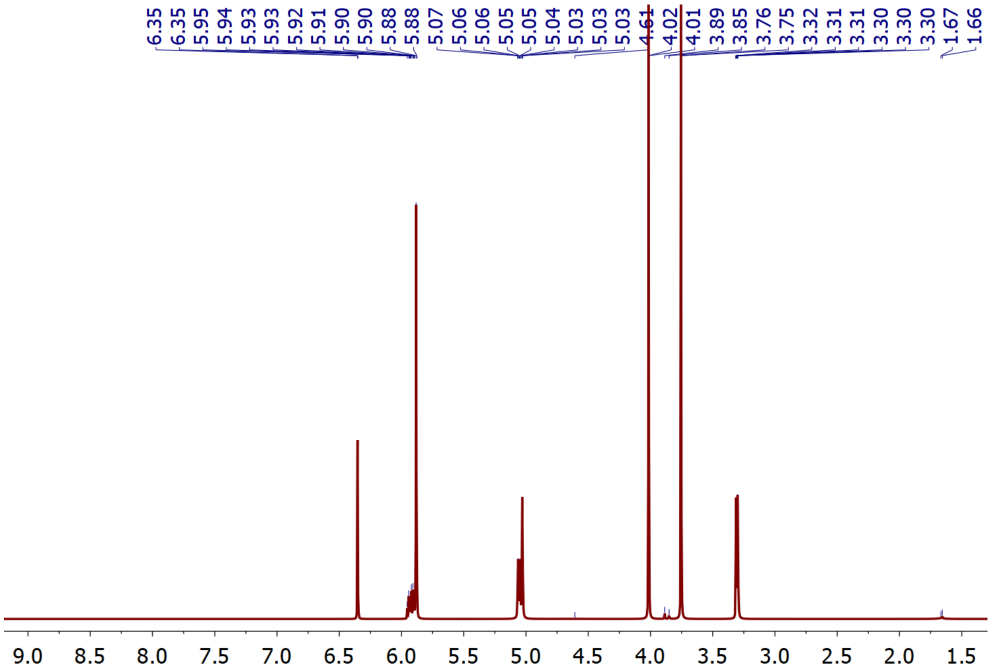

(a)

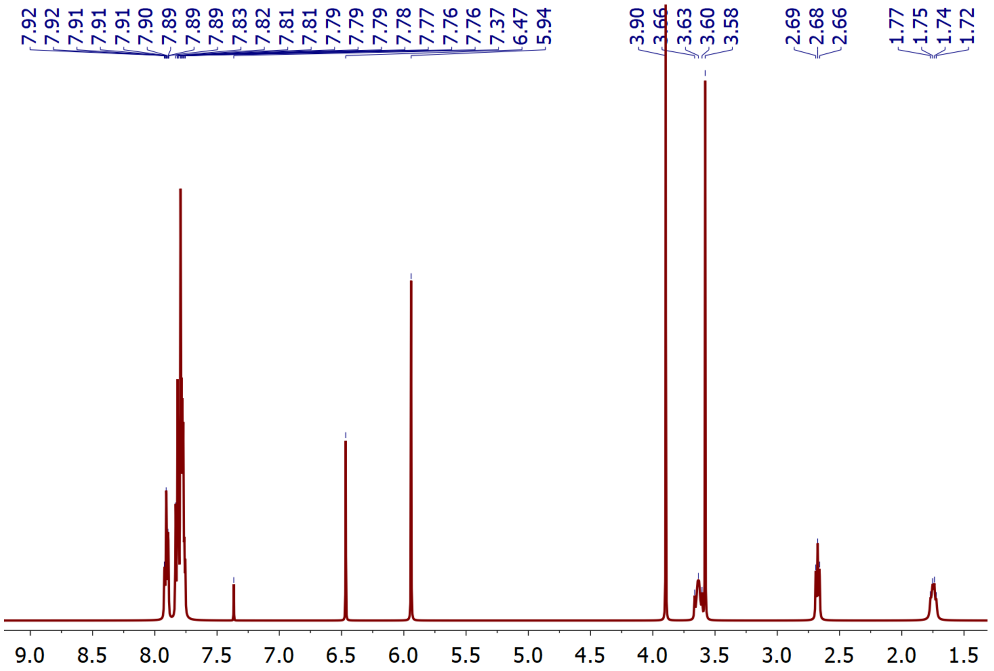

(b)

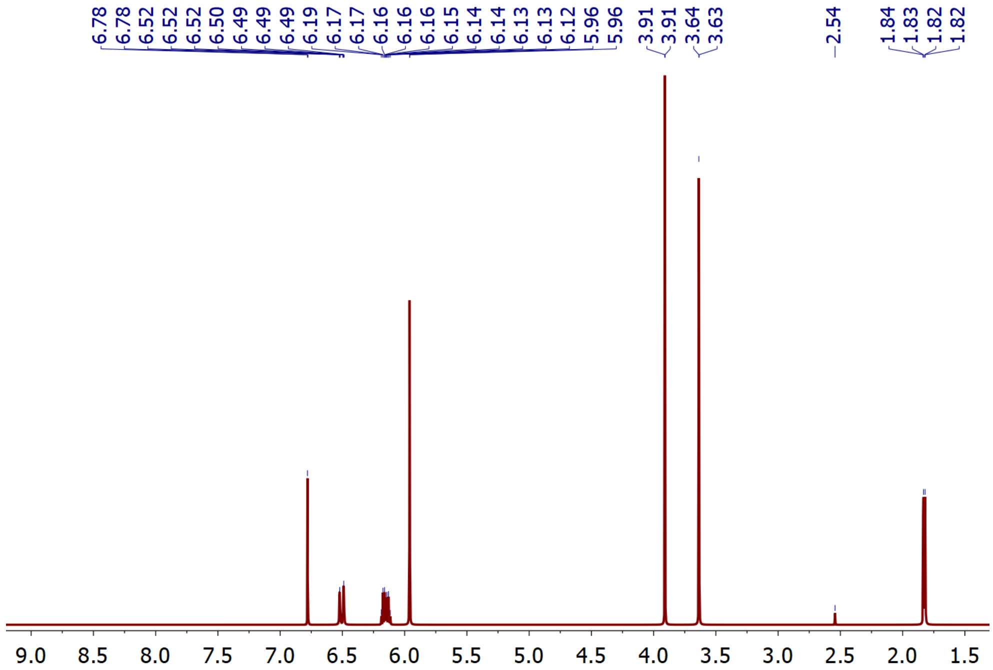
6.78 6.78 6.52 6.52 6.52 6.50 6.49 6.49 6.49 6.19 6.17 6.17 6.16 6.16 6.15 6.14 6.14 6.13 6.13 6.12 5.96 5.96 3.91 3.91 3.64 3.63 2.54 1.84 1.83 1.82 1.82
9.0 8.5 8.0 7.5 7.0 6.5 6.0 5.5 5.0 4.5 4.0 3.5 3.0 2.5 2.0 1.5

(c)

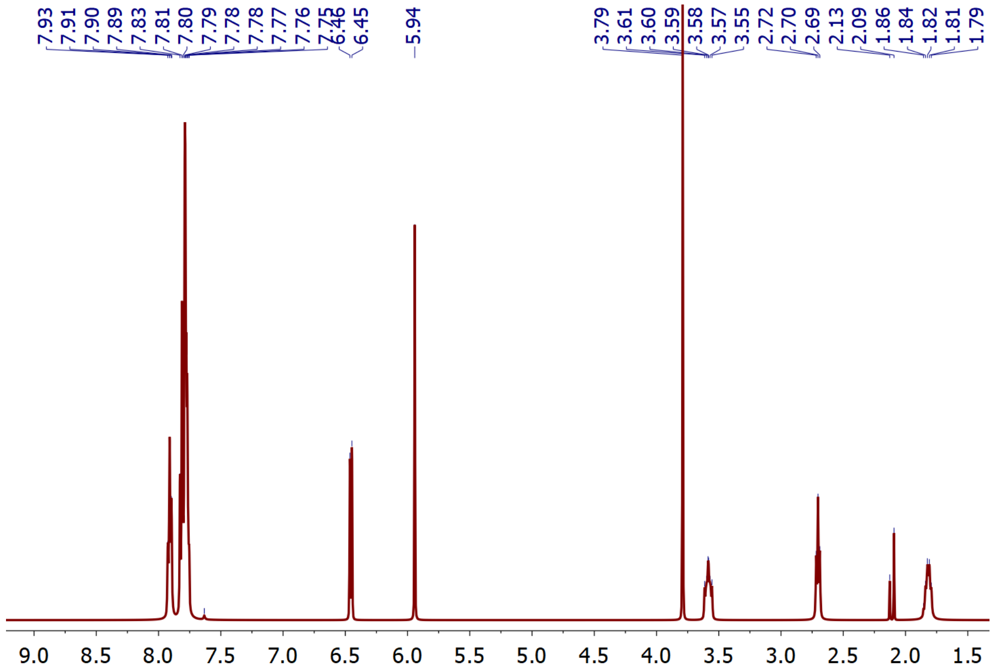
7.93 7.91 7.90 7.89 7.83 7.81 7.80 7.79 7.78 7.78 7.77 7.76 7.75 6.46 6.45 5.94 3.79 3.61 3.60 3.59 3.58 3.57 3.55 2.72 2.70 2.69 2.13 2.09 1.86 1.84 1.82 1.81 1.79
9.0 8.5 8.0 7.5 7.0 6.5 6.0 5.5 5.0 4.5 4.0 3.5 3.0 2.5 2.0 1.5

(d)
